# Rhinal and hippocampal event-related potentials as epileptogenic zone markers in the pre-surgical evaluation of temporal epilepsies: a systematic review

**DOI:** 10.1055/s-0043-1761493

**Published:** 2023-05-31

**Authors:** Daniela de Andrade Morange, Magali Teresópolis Reis Amaral, Martha Silvia Martinez-Silveira, Agnès Trébuchon

**Affiliations:** 1 Institut de Neurosciences des Systèmes, Université d'Aix-Marseille, Marseille, France.; 2 Hospital Universitário Prof. Edgard Santos, Departamento de Neurofisiologia Clínica, Salvador BA, Brazil.; 3 Universidade Estadual de Feira de Santana, Departamento de Ciências Exatas, Salvador BA, Brazil.; 4 Fundação Oswaldo Cruz, Instituto Gonçalo Muniz, Salvador BA, Brazil.; 5 Neurophysiologie Clinique, Assistance Publique - Hôpitaux de Marseille, Hôpital de la Timone, Marseille, France.

**Keywords:** Evoked Potentials, Epilepsy, Temporal Lobe, Memory, Perirhinal Cortex, Hippocampus, Potenciais Evocados, Epilepsia do Lobo Temporal, Memória, Córtex Perirrinal, Hipocampo

## Abstract

**Background** Cognitive event-related potentials (ERPs) allow for lateralization of the epileptogenic zone (EZ) to estimate the reserve of memory in the contralateral non-epileptogenic hemisphere, and to investigate the prognosis of temporal lobe seizure control in unilateral temporal lobe epilepsy (TLE).

**Objective** To define the accuracy of cognitive evoked anterior mesial temporal lobe (AMTL-N400) and P600 potentials in detecting the epileptogenic zone in temporal lobe epilepsy (TLE), and second, to evaluate the possibility of using them as markers of cognitive outcome.

**Methods** The systematic review using Medline/PubMed, Embase, and Lilacs database was conducted in September 2021. Only articles published in English from 1985 to June 2021 were included. We searched for studies with: (1) depth intracranial electroencephalography (iEEG) recordings analysis of rhinal and hippocampal activity (2) correlations between ERP results obtained in the mesial temporal regions (AMTL-N400 and P600) and the epileptogenic zone.

**Results** Six out of the seven studies included in this review defined the laterality of the epileptogenic zone (EZ) during presurgical investigation using ERPs. One study showed that the contralateral AMTL-N400 predicts seizure control. Another study found correlation between the amplitudes of the right AMTL-N400 and postoperative memory performance.

**Conclusions** There is evidence that the reduced amplitude of the AMTL-N400 has high accuracy in identifying the epileptogenic zone, as it does in estimating the extent of seizure control and memory impairment in postoperative patients.

## Resumo

**Antecedentes** Potenciais relacionados a eventos (PREs) cognitivos permitem a lateralização da zona epileptogênica (ZE), estimar a reserva de memória no hemisfério contralateral não-epileptogênico, e estimar o prognóstico pós-operatório em pacientes com epilepsia do lobo temporal (ELT) unilateral quanto ao controle de crises.

**Objetivo** Definir a acurácia dos potenciais evocados cognitivos do lobo temporal mesial anterior (LTMA-N400) e P600 na detecção da zona epileptogênica na epilepsia do lobo temporal (ELT), além de avaliar a possibilidade de usá-los como marcadores de desfecho cognitivo.

**Métodos** A revisão sistemática foi realizada em setembro de 2021 usando as bases de dados Medline/PubMed, Embase e Lilacs. Apenas artigos publicados em inglês no período entre 1985 e junho de 2021 foram incluídos. Buscamos estudos com: (1) análises dos registros de electroencefalografia intracraniana (EEGi) da atividade rinal e hipocampal (2) correlações entre os resultados de PREs obtidos nas regiões temporais mesiais (AMTL-N400 e P600) e a zona epileptogênica.

**Resultados** Seis dos sete estudos incluídos nesta revisão definiram a lateralidade da zona epileptogênica (ZE) durante a investigação pré-cirúrgica usando PREs. Um estudo mostrou que o AMTL-N400 contralateral prediz o controle das crises. Outro estudo encontrou correlação entre as amplitudes do AMTL-N400 direito e o desempenho da memória pós-operatória.

**Conclusões** Há evidências de que a amplitude reduzida do AMTL-N400 tem alta precisão na identificação da zona epileptogênica, assim como na estimativa do prognóstico quanto ao controle de crises a longo prazo e prejuízo da memória em pacientes submetidos à cirurgia ressectiva.

## INTRODUCTION

The anterior mesial temporal lobe (AMTL-N400) 1 and P600 2 cognitive event-related potentials (ERPs) are generated in the rhinal and hippocampal cortex, respectively. Using intracranial electrodes, cognitive ERPs are measured to analyze the brain's response to stimuli associated with the recognition of familiar and new words or pictures, and through more complex tasks during memory formation. The present study is a review of the articles that analyze this as a tool to locate the epileptogenic zone.

Temporal lobe epilepsy (TLE) is the most frequent form of focal epilepsy in adult patients. 3 The mesial temporal lobe (MTL) is often investigated as part of the epileptogenic zone in surgical candidates, allowing for an investigation of structures such as the ento- and perirhinal cortexes and the hippocampus. It is well known that the medial temporal lobe (MTL) is involved in memory formation and the hippocampus in the information storage process. 4 Thus, surgery for TLE can be related to memory impairment. 5 The preservation of the functionality of the temporal mesial structures is evaluated with preoperative investigations, using structural and functional methods. In this context, the study of the AMTL-N400 and P600 potentials during stereo-EEG investigations has been proposed, allowing us to analyze the preservation of the functionality of the rhinal and hippocampal cortexes, where these potentials are generated.

Grunwald et al. 6 used a word recognition paradigm and recorded the entorhinal and hippocampal ERPs elicited by words identified as either *new* or *old* . They compared the ERPs elicited by *old* words that were correctly recognized as repetitions, with those that were not. They found that AMTL-N400 amplitudes reduced with repetition ( *old* words), independent of whether these repetitions were recognized as such or not. This suggests that the entorhinal cortex is involved in the recognition process, independent of a person's awareness of recognition. Furthermore, they found a marked hippocampal late component (P600) only elicited by repetitions when these were consciously recognized. This suggests that the hippocampus proper is involved in the conscious memory process.

In some studies, 7 8 9 there was an association between diminished AMTL-N400 and the side of the hippocampal pathology. It was found that the AMTL-N400 to first presentations of words ( *new* ) and *new* -minus- *old* repetition effects were diminished on the side of the hippocampal pathology. Grunwald et al. 10 concluded that when the repetition effects are reduced contralateral to the side of hippocampal sclerosis, this may indicate bilateral epileptogenicity, indicating risk of seizure recurrence after the operation. Previous studies 11 found that P600 showed higher amplitudes for word repetitions than for first presentations ( *new* ), but because this was not reproducible in all patients, it was not taken into account for focus lateralization.

Preoperative markers of hippocampal functionality (neuropsychological test scores and contralateral intracarotid amytal procedure (CIAP) memory test of the surgical hemisphere) and structural measurements (hippocampal cell densities and MRI volumes) predict postoperative memory decline, consistent with the functional adequacy/functional reserve mode. 12 13 14 15 Based on hippocampal neuronal loss, the greater the degree of the pathology in the left epileptogenic hippocampus, the smaller the impact that surgical resection will have on postoperative verbal memory performance. 16 Kneebone et al. 17 showed the importance of the CIAP memory test as an index of the functional capacity of the temporal lobe being considered for surgery, and to predict postoperative memory changes, as measured with the Wechsler memory scale-revised (WMS-R). Patients with LTE on the left side, who had good memory function as recorded with the CIAP, tended to have a greater decrease in postoperative verbal memory. Then, there was an inverse correlation between the CIAP scores and postoperative verbal memory.

Baxendale et al. 18 identified variables that they considered to be predictors of higher risk of cognitive loss in the postoperative period, in patients with left mesial temporal epilepsy. These variables were: higher neuropsychological test scores associated with structures ipsilateral to the area to be operated on (verbal learning scores), and lower scores associated with contralateral structures (visual learning scores). In accordance with the findings of the neuropsychological evaluation, the preoperative MTL functionality markers (AMTL-N400 and P600) would allow us to assess the risk of resection of the rhinal and hippocampal cortex, causing functionality loss.

Hence, a review article is relevant to better identify the effectiveness of this marker in defining the epileptogenic zone and preserving the functionality of the hippocampus and rhinal cortexes. In this systematic review, the population of interest is composed of patients with drug-resistant TLE, candidates for epilepsy surgery, investigated through intracranial electroencephalography (iEEG) with electrodes in the rhinal and hippocampal cortexes, and the *new* - *old* paradigm for detection of AMTL-N400 and P600. When the study performed a correlation test between the power spectra, amplitudes, or latencies of the ERPs on the side of the epileptogenic focus in relation to the non-epileptogenic side, including or not analyses of memory test results, it was included in our review. The main parameter of interest is to define the accuracy of AMTL-N400 and P600 in detecting the epileptogenic zone. And second, to correlate AMTL-N400 and P600 ERPs to verbal memory test results.

## METHODS

A preferred reporting items for systematic reviews and meta-analyses (PRISMA) checklist 19 was followed to produce a systematic review, and the protocol was registered on the PROSPERO website under number CRD42020189309.

The search and selection of the studies were performed according to the PICOS strategy:

- Population - patients with focal drug-resistant epilepsy.
- Intervention/exposure - patients submitted to an iEEG, investigating the hippocampus in the preoperative phase, and undergoing the recognition memory paradigm.
- Comparison – hippocampal AMTL-N400/P600 of the epileptogenic focus versus the AMTL-N400/P600 response of the contralateral hippocampus.
- Outcome – studies that evaluate AMTL-N400/P600 as a marker of the epileptogenic zone.
- Study type – descriptive studies, cohort, and cross-sectional studies.

The question of the systematic review was: what is the accuracy of the cognitive evoked potential AMTL-N400/P600 in detecting the epileptogenic zone in TLEs?

### Searching the literature

The bibliographic searches were done in September 2021 using the Medline/PubMed, Embase, and Lilacs databases, a manual search in the review article references for potentially eligible articles, track references for selected articles, and a complementary search on Google Scholar.

The keywords used were intracranial electroencephalography, iEEG, evoked potentials, event-related potentials, N400, P600, hP600, ERP, and AMTL-N400. The search was done systematically in the same way for both online databases.

### MedLine/PubMed search strategy

1°: Electroencephalography (MeSH terms) OR brain mapping (MeSH terms) OR intracranial electroencephalography OR iEEG OR SEEG OR intracranial EEG OR stereo-electroencephalography OR stereo-EEG OR stereoelectroencephalography.
2°: (evoked potentials [Mesh] OR event-related potential* OR evoked potential* OR “ERP) AND (N400 OR P600 OR hP600 OR AMTL-N400 OR new-old OR recognition task OR memory task).
3°: epilepsy [MeSH Terms] OR epilepsy* [TW] OR hippocampus OR temporal lobe epilepsy OR epileptic zone;
4°: 1° and 2° and 3°.

### Eligibility criteria and study selection

We included articles published in English from 1985 to June 2021. Observational studies in humans considered for inclusion were case series, cohort studies, and cross-sectional studies. The identified titles, abstracts, and full-text articles were read and blindly selected by two independent reviewers. In the case of disagreement, a third reviewer resolved any possible controversy.

We excluded articles from before 1985 as these use a different methodology as well as studies that used surface EEG recordings and, instead, concentrated our research on studies that used iEEG recordings. Studies that did not correlate AMTL-N400 to the EZ were also excluded.

Finally, when we found articles by the same author on AMTL-N400 and the laterality on the epileptogenic zone, referring to studies with the same outcome, we excluded the article with the lowest number of patients included in the study.

### Assessment of methodological quality

We applied the Joanna Briggs Institute (JBI) 20 scale to cross sectional studies to evaluate them in terms of methodological quality, since only this type of methodology was found in the articles. The scale consists of eight items. The analysis was done by two reviewers, and disagreements were resolved by a third examiner.

### Data collection and analysis

Data from the articles was extracted and classified using a data extraction Excel spreadsheet (Microsoft Corp., Redmond, WA, USA). Classification was determined based on what was considered relevant data at the beginning of the bibliographical study and improved upon, according to the inclusion of further relevant data from the articles. Two independent researchers read the selected studies and extracted the data in pairs.

Analysis of the results was based on agreement between the abnormal response of the AMTL-N400/P600 in the memory paradigm and the epileptogenic zone, defined through clinical and electrographic diagnosis, using recordings with depth iEEG, considered the gold-standard method in patients with unilateral TLE. Sensitivity of the method to separately define the laterality of the epileptogenic zone in TLE, as well as the presence or absence of hippocampal sclerosis, was investigated and discussed in the articles.

## RESULTS

### Research results

**Figure 1:**
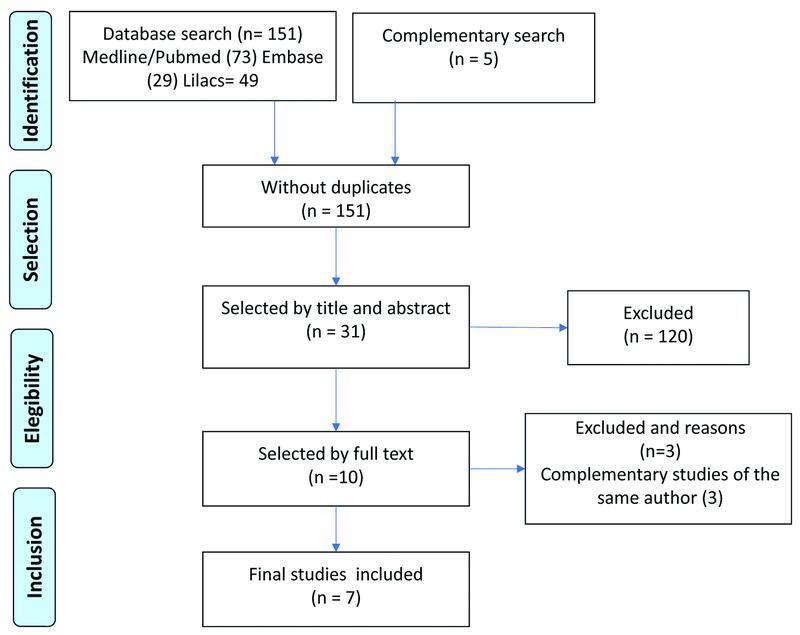
Flowchart of the studies search and selection process.

The bibliographic search resulted in 73 citations from the Medline/PubMed databases and 29 citations from Embase and 49 from Lilacs, with 5 studies being duplicated ( Figure 1 ). A complementary search on Google Scholar and references of the selected articles was done, after which 5 articles were found. Finally, 151 studies were submitted to a selection process based on the title and abstracts, and 31 of them were excluded from full-text reading. The full text of the 10 selected citations has been examined in more detail. Three of these were then excluded because they were complementary studies from the same group, Guillem et al., 21 Elger et al. 7 and Grunwald et al. 8 Two articles, by Guillem et al. 21 and Guillem et al., 22 of the same authorship were about complementary studies, so we only included the article 23 with the greater number of patients included in the study. Similarly, the two other articles, by Grunwald et al. 8 and Elger et al., 7 were excluded, as they were about the same topic and had the same outcome as another study by Grunwald et al., 9 but used a smaller number of patients.

#### Description of included studies

Seven 9 10 22 24 25 26 27 studies were chosen because they met the inclusion criteria ( Table 1 ). The included articles were published between 1998 and 2008; the majority (n 6/7) were European studies, and one (n 1/7) was an Australian study. 27 The study design of all articles was cross-sectional, and the population studied in the selected articles was between 9 and 70 patients. A total of 238 patients were studied, 218 of which had unilateral TLE.

**Table 1:** Description of the methods applied in the included studies

Authors	Study design	Population	iEEG method	Recognition task
Puce et al., 1991 27	Cross-sectional study	20 patients (16 with unilateral TLE)	Bilateral iEEG using an orthogonal approach with the anterior hippocampus as the target.	Verbal and visuospatial memory tasks
Guillem et al., 1998 22	Cross-sectional study	25 patients (9 with unilateral TLE)	iEEG was used to approach the targets (Hp, Amg, LTc) from the lateral skull surface. Electrodes were implanted unilaterally or bilaterally.	Recognition memory task for verbalizable pictures
Grunwald et al., 1998a 9	Cross-sectional study	50 patients with unilateral TLE (29 with HS)	Bilateral iEEG inserted along the longitudinal axis of the hippocampus with the amygdala as the target.	Word recognition paradigm (new/old)
Grunwald et al., 1998b 25	Cross-sectional study	40 patients with left TLE	Bilateral iEEG, inserted along the longitudinal axis of the hippocampus, with the amygdala as the target.	Word recognition paradigm (new/old)
Grunwald et al., 1999 10	Cross-sectional study	70 patients with unilateral TLE (67 with HS)	Bilateral iEEG, inserted along the longitudinal axis of the hippocampus, with the amygdala as the target.	Word recognition paradigm (new/old)
Mormann et al., 2007 26	Cross-sectional study	9 patients with unilateral TLE and HS	Bilateral iEEG, inserted along the longitudinal axis of the Hp with the parahippocampal gyrus as the target.	Word memorization paradigm (new/old)
Dietl et al., 2008 24	Cross-sectional study	24 patients with unilateral mesial TLE and HS	Bilateral iEEG, inserted along the longitudinal axis of the Hp, with the parahippocampal gyrus as the target.	Verbal and pictorial recognition paradigm

Abbreviations: Amg, amygdala; Hp, hippocampus; HS, hippocampal sclerosis; LTc, lateral temporal cortex.

#### EEG recording

In most of the studies, the patients were investigated via bilateral analysis of the iEEG. Only one study 25 included patients investigated unilaterally. Event-related potentials were recorded by electrodes implanted in different ways. Puce et al. 27 and Guillem et al. 22 used an orthogonal approach, from the lateral skull surface, while Grunwald et al., 9 10 25 Morman et al. 26 and Dietl et al. 24 implanted electrodes along the longitudinal axis of the hippocampus.

Amplitude and latency were determined. In 6 studies, the ERPs peak amplitudes were measured with respect to a 200 milliseconds prestimulus baseline, and, in one study, Puce et al., 27 to a 500 milliseconds prestimulus. Event-related potential waveforms were inspected visually; the amplitude was chosen with the maximum ERP peak-amplitude, occurring in the latency range of 300 to 500 milliseconds for AMTL-N400, and 500 to 1,200 milliseconds for P600.

In all the analyzed research, patients were only included in the study if ERP data was found by visual inspection, so as not to be contaminated by epileptic discharges.

#### Patients

Four studies, by Grunwald et al., 25 Grunwald et al., 10 Morman et al., 26 and Dietl et al. 24 analyzed only patients with hippocampal sclerosis, comparing the responses of ipsilateral and contralateral ERPs to those of the epileptogenic zone, including a total of 140 patients. All these four articles evaluated the AMTL-N400 response to lateralizing the EZ and found a significant drop in amplitude and reduced repetition effect on the side of the EZ.

Grunwald et al. 9 compared the AMTL-N400 generated in the group with hippocampal sclerosis (HS) in 29 patients, and in the group with EZ without HS in 21 patients. Two studies, by Puce et al. 27 and Guillem et al., 22 using a total of 25 patients, did not analyze HS patients separately.

Regarding gender distribution, 89 women and 129 men were included, with an age range of 13 to 54 years. Age and EZ distribution are described in Table 2 .

**Table 2:** Classification of patients as to epileptogenic zone (left/right TLE), gender, and age range distribution

Author	Population	Age range/years	Mean age/years	TLE
Puce et al., 1991 27	7 women and 9 men	16–50	32.4 ± 8.6	9 left 7 right
Guillem et al., 1998 22	3 women and 6 men	18–36	27.8 ± 5.9	3 left 6 right
Grunwald et al., 1998a 9	20 women and 30 men	13–51	not informed	27 left, 23 right
Grunwald et al.,1998b 25	18 women and 22 men	14–52	32 ± 11	40 left
Grunwald et al., 1999 10	30 women and 40 men	16–51	33.3	27 left 43 right
Mormann et al., 2007 26	6 women and 3 men	not informed	34.1 ± 8.3	3 left 6 right
Dietl et al., 2008 24	5 women and 19 men	20–54	34 ± 9	12 left 12 right

Abbreviation: TLE, temporal lobe epilepsy.

#### Verbal versus pictorial recognition tasks

Three studies, by Puce et al., 27 Guillem et al., 22 and Dietl et al., 24 described the AMTL-N400/P600 responses in the EZ and contralateral to the EZ, analyzing pictorial recognition tasks. In two studies, by Puce et al. 27 and Guillem et al., 22 a total of 45 patients were studied, 25 of whom had unilateral TLE. They did not classify them according to HS occurrence.

Puce et al. 27 studied nonverbal (visuospatial) recognition memory tasks, using abstract nonverbalizable stimuli eliciting ERPs of similar morphology to their verbal analogues. They showed that AMTL-N400/P600 were either present bilaterally or absent bilaterally and, therefore, failed to find the epileptogenic focus.

In a study by Guillem et al., 22 the stimuli were 240 verbalizable pictures of common objects. The word recognition memory task was not applied. They concluded that the modulation of the P600 component seems to be less reliable, and that the AMTL-N400 is the most valuable ERP index for investigating functioning temporal lobe structures.

Dietl et al. 24 studied 24 patients in whom HS was confirmed by word recognition and pictorial recognition tasks. They concluded that limbic ERPs to words are more sensitive to the epileptogenic process than those to pictures. Then, both studies, by Guillem et al. 22 and Dietl et al., 24 concluded that pictorial recognition paradigms are less sensitive to the EZ, and Dietl et al. 24 showed that the verbal paradigm is more accurate in detecting the EZ than the pictorial task. All the other four analyzed research studies used only word recognition tasks.

The quality of the studies included in this review was evaluated using the JBI scale ( Table 3 ), according to which high methodological quality was observed.

**Table 3:** Methodological classification assessed with the JBI scale for cross sectional studies

Authors/year	Criteria								
	1	2	3	4	5	6	7	8	Total
Puce et al., 1991 27	N	Y	Y	Y	N	NA	Y	Y	5
Guillem et al., 1998 22	N	Y	Y	Y	N	NA	Y	Y	5
Grunwald et al., 1998a 9	Y	Y	Y	Y	N	NA	Y	Y	6
Grunwald et al.,1998b 25	N	Y	Y	Y	N	NA	Y	Y	5
Grunwald et al., 1999 10	Y	Y	Y	Y	N	NA	Y	Y	6
Mormann et al., 2007 26	N	Y	Y	Y	N	NA	Y	Y	5
Dietl et al., 2008 24	N	Y	Y	Y	N	NA	Y	Y	5

Abbreviations: N, no; NA, not applicable; Y, yes.

Notes: 1. criteria for inclusion in the sample defined; 2. Subjects and the setting described; 3. Exposure measured in a valid way; 4. Standard criteria for measurement of the condition; 5. Confounding factors identified; 6. Strategies to deal with confounding factors stated; 7. Outcomes measured in a valid way; 8. Appropriate statistical analysis.

The main characteristics of the outcomes of the articles are described in Table 4 .

**Table 4:** Description of outcomes in included studies

Authors	Results to lateralization EZ		Good marker	Results in performance
	AMTL-N400	P600		
Puce et al., 1991 27	N400 amplitude for each stimulus type indicated N400 amplitude was larger in response to novel stimuli and smaller in response to repeated stimuli. N400/P600s were either present bilaterally or absent bilaterally, and thus were not of value in localizing the epileptogenic focus.	P600 amplitude data indicated that repeated stimuli elicited larger P600s. N400/P600s were either present bilaterally or absent bilaterally, and thus were not of value in localizing the epileptogenic focus.	No	NA
Guillem et al., 1998 22	The AMTL- N400 is usually less negative when evoked by “old” rather than by “new” words in the oddball tasks. The AMTL-N400 amplitude is altered by the presence of the epileptic zone.	The modulation of the P600 component seems to be less reliably observed, at least in the iEEG	Yes	NA
Grunwald et al., 1998a 9	In patients with HS, AMTL-N400 to first presentation of new words were reduced on the side of epileptogenic focus and no repetition effects were found. In patients without HS AMTL-N400 amplitudes to new and old items were smaller in the epileptic temporal lobe, and repetition effects were found.	NA	Yes	The number of correctly identified first representations and repetition was correlated only with left AMTL-N400 amplitude to first presentation (new), in preoperative patients.
Grunwald et al., 1998b 25	Upon repetition, AMTL-N400 was reduced in amplitude only on the non-epileptogenic side ( *p* < 0.0005). On the epileptogenic side repetition effects (new-minus-old) were reduced.	NA	Yes	The amplitudes of the right AMTL-N400 to new words correlated with the number of words recalled after a 30-minute delay. The amplitudes of the right AMTL-N400 to new words correlated with the percentage of postoperative change, relative to the preoperative performance.
Grunwald et al., 1999 10	Seizure freedom was associated with significantly higher values for new-minus-old amplitude differences on the non-lesioned side. The repetition effect permitted correct prediction of postoperative seizure control in 94% of all patients. AMTL-N400 to new words and repetition effects were diminished on the side of HS. In patients without HS, AMTL-N400 amplitude to new and old words were smaller in the epileptic lobe, and the repetition effect is present.	NA	Yes	NA
Mormann et al., 2007 26	Decreased ERP (AMTL-N400) amplitudes on the ipsilateral side, with hippocampal sclerosis, and a significant decrease of stimulus-induced power in the delta and theta range on the side of hippocampal sclerosis.	To a lesser extent for the hippocampal P600 on the ipsilateral side, with hippocampal sclerosis, in comparison with the contralateral side.	Yes	Increased amplitudes for subsequently remembered versus forgotten words showed a significant decrease in delta and theta range power on the hippocampus and the rhinal cortex on the ipsilateral side.
Dietl et al., 2008 24	AMTL-N400s to *new* word and not to pictures were reduced in amplitude near the epileptogenic focus, both in right and in left TLE. Limbic ERPs to words are more sensitive to the epileptogenic process than those to pictures.	LNCs to *old* word and pictures were smaller in sclerotic than in non-sclerotic hippocampi.	Yes	NA

Abbreviations: AMTL-N400, anterior mesial temporal lobe; iEEG, intracranial electroencephalography; ERP, Event-related potential; EZ, epileptogenic zone; HS, hippocampal sclerosis; NA, not applicable, analysis not performed; TLE, temporal lobe epilepsy.

### Main results

#### AMTL-N400 and the epileptic temporal lobe

The study by Puce et al., 27 using verbal and visuospatial (nonverbal) tasks, showed that AMTL-N400 was larger to new stimuli, whereas P600 was larger to repeated stimuli, in agreement with the publications that followed. However, they found that AMTL-N400/P600 were present bilaterally or absent bilaterally and, therefore, failed to find the epileptogenic focus ( Table 3 ). Among the 20 patients investigated, in AMTL-N400 was present bilaterally in 17 and absent in 3 patients. 27

In a study by Grunwald et al., 9 the patients were investigated by bilateral depth electrodes implanted stereotaxically along the longitudinal axis of the hippocampus, with the amygdala as the target of the most anterior contact. Maximal AMTL-N400 to words were recorded anterior to the hippocampus proper and near the amygdala. Findings from previous publications by Grunwald et al. 8 and Elger et al. 7 were confirmed, but with a larger number of patients: 29 patients with HS and 21 with extrahippocampal lesions. They showed that (i) the AMTL-N400 to first presentation of a word ( *new* ) were reduced on the epileptogenic side ipsilateral to HS; (ii) in patients with HS, the repetition effects ( *new* -minus - *old* ) were found only on the contralateral side, and AMTL-N400 amplitudes to *old* words were higher ipsilateral to HS than on the contralateral side; (iii) in patients without HS, the AMTL-N400 amplitudes to *new* and *old* words were smaller in the ETL, and the *new* -minus- *old* repetition effect was found; (iv) the number of correctly identified words was correlated only with the left AMTL-N400 amplitude to *new* stimuli, in a preoperative memory test.

Grunwald et al. 10 investigated 70 patients confirming the findings of previous studies that AMTL-N400 amplitudes to *new* words is reduced on the HS side, and repetition effects are diminished on the side of HS. They showed that seizure freedom is associated with significantly higher values for *new* -minus- *old* amplitude differences (repetition effects) on the non-lesioned side. The presence of a contralateral AMTL-N400 for *new* -minus- *old* effect predicts postoperative seizure control in 94% of all patients.

Dietl et al., 24 studied 24 patients in whom HS was confirmed by verbal recognition and pictorial recognition tasks. Bilateral depth electrodes were implanted stereotactically along the longitudinal axis of the hippocampus from an occipital approach with the rhinal cortex, that is, the medial temporal cortex anterior to and just below the amygdala, as the target for the most anterior electrode. The AMTL-N400 to *new* words and the late negative component (LNC) to *old* words were reduced in amplitude on the side of seizure origin. They concluded that the AMTL-N400 and LNC-amplitudes to pictures led to wrong lateralizations (17%) and that the same analysis, based on limbic ERPs to words, classified all patients correctly, with words rather than pictures being more sensitive to the epileptogenic process.

Mormann et al. 26 studied nine patients investigated by bilateral depth electrodes implanted stereotaxically along the longitudinal axis of the hippocampal formation, with the anterior contacts in the parahippocampal gyrus, which is covered by the rhinal cortex, and the posterior contacts located within the hippocampus. They described a decreased AMTL-N400 and, to a lesser extent, a decreased hippocampal LPC/P600 on the side of HS. They confirmed the findings of Fernández et al. 23 by showing a positive correlation between the increased size of the AMTL-N400 and hippocampal LPC and the remembered words, but not forgotten words, suggesting that successful memory formation is accompanied by a significant increase in the rhinal AMTL-N400 and the hippocampal LPC. They showed another important finding, significantly decreased ERP amplitudes on the side of HS are caused by a decrease in the delta and theta power observed in the hippocampus and the rhinal cortex. They suggest that this decrease in power probably reflects a lower number of neural assemblies that are recruited into the memorization task.

#### ERPs and verbal memory performance

Grunwald et al. 25 investigated 40 patients with left TLE before and after surgery, with all patients undergoing resection of the medial temporal structures. The patients were investigated by bilateral depth electrodes implanted stereotaxically along the longitudinal axis of the hippocampus with the amygdala as the target of the most anterior contact. They confirmed the findings that repetition effects were preserved only on the non-epileptogenic side. They showed new findings: (i) the amplitudes of the right AMTL-N400 (non-epileptogenic side) to *new* words correlated with the numbers of words recalled after a 30 minutes delay ( *p* < 0.0005) in preoperative performance; (ii) the amplitudes of the right AMTL-N400 (non-epileptogenic side), but not the left AMTL-N400, to *new* words correlated with the percentage of postoperative change relative to the preoperative performance ( *p* < 0.00005). They concluded that the greater the functional integrity of the right MTL-structures, the more likely these are to compensate for the loss of functionality of the removed left MTL-structures. They could, thus. predict the postoperative verbal recall performance. Additionally, they did not find a correlation, but they described in a multiple regression analysis that the larger the AMTL-N400 in the left hemisphere preoperatively, the greater the drop in postoperative free recall performance, showing that the effects of surgically removing the left MTL-structures are more marked the better they were functioning preoperatively.

## DISCUSSION

For many patients with refractory focal epilepsies, it is necessary to insert bilateral depth electrodes to define the EZ when non-invasive studies have remained non-conclusive to lateralize the zone involved at the onset of the seizures. In these cases, ERPS proved to be a way to improve this analysis, both confirming the findings of lateralization of the EZ and also informing about the functional integrity of these mesial structures that participate in the memory process. Six of the studies 9 10 22 24 25 26 in our review showed an association between reduced AMTL-N400 and the EZ. Puce et al. 27 did not describe reduced AMTL-N400 to *new* stimuli or reduced repetition effects in the EZ.

The result of the systematic review showed that the reduced AMTL-N400 amplitude to *new* words in a word recognition paradigm have high accuracy in detecting the laterality of the EZ in unilateral TLEs. 9 25 Dietl et al. 24 showed that the verbal paradigm is more accurate than the pictorial task in detecting the EZ. Besides, the absence of AMTL-N400 on contralateral mesial structure have a high accuracy to rule out the diagnosis of secondary epileptogenicity. 10

A high sensitivity of reduced AMTL-N400 to lateralize the EZ was found in hippocampi with HS in five studies. 9 10 24 25 26 In a study by Grunwald et al., 9 correlation was shown between a reduced AMTL-N400 amplitude to *new* and *old* words and the EZ in the group without HS. 9 We believe that an analysis of a larger sample of patients without HS is necessary to better represent different severities of TLE and to analyze the usefulness of this method in detecting hippocampal dysfunction without histological injury.

Each of the seven articles included in this review clearly described the inclusion criteria and objective of the study, and used an appropriate methodology to investigate and quantify the results. Six of these articles showed a good accuracy of AMTL-N400s to *new* words in detecting the EZ in unilateral TLE. Among the 7 studies that met the selection criteria to answer our main question whether AMTL-N400/P600 is a good marker of the EZ, 1 provided information regarding the correlation between verbal postoperative performance and the rhinal evoked potential. In a study by Grunwald et al., 25 the findings indicate that the greater the functional integrity of the right rhinal cortex, the more likely compensation will occur for the loss of functionality with the resection of the mesial structures of the left temporal lobe.

Three studies analyzed performance during the tasks. To answer our question of whether AMTL-N400/P600 is a good marker of postoperative verbal memory performance, only 1 study 25 investigating patients with HS showed that the amplitudes of the right AMTL-N400 (non-epileptogenic side) to *new* words correlated with the number of words recalled after a 30-minute delay.

Due to the importance of functional analysis of the hippocampus and rhinal cortexes, further studies are needed to clarify the correlation between ERP amplitude measurements, including in cases in which there is no HS, but only cortical dysfunction due to epileptogenicity. This analysis of ERPs would elucidate the interrelationship between the specialized language hemisphere and the contralateral cortex in the storage of different information modalities.
